# Regulation of the Hippocampal Network by VGLUT3-Positive CCK- GABAergic Basket Cells

**DOI:** 10.3389/fncel.2017.00140

**Published:** 2017-05-16

**Authors:** Caroline Fasano, Jill Rocchetti, Katarzyna Pietrajtis, Johannes-Friedrich Zander, Frédéric Manseau, Diana Y. Sakae, Maya Marcus-Sells, Lauriane Ramet, Lydie J. Morel, Damien Carrel, Sylvie Dumas, Susanne Bolte, Véronique Bernard, Erika Vigneault, Romain Goutagny, Gudrun Ahnert-Hilger, Bruno Giros, Stéphanie Daumas, Sylvain Williams, Salah El Mestikawy

**Affiliations:** ^1^Douglas Mental Health University Institute, Department of Psychiatry, McGill University, MontrealQC, Canada; ^2^Institut für Integrative Neuroanatomie Research CenterBerlin, Germany; ^3^Sorbonne Universités, UPMC Univ Paris 06, Centre National de la Recherche Scientifique, Institut National de la Santé et de la Recherche Medicale, Institut de Biologie Paris Seine, Neuroscience Paris Seine (NPS)Paris, France; ^4^Université Paris Descartes, Sorbonne Paris Cité, Centre National de la Recherche Scientifique, UMR 8250Paris, France; ^5^OramacellParis, France; ^6^Sorbonne Universités, UPMC Univ Paris 06, CNRS, Core Facilities - Institut de Biologie Paris SeineParis, France; ^7^CNRS UMR 7364, Team NCD, Université de StrasbourgStrasbourg, France

**Keywords:** hippocampus, basket cell, vesicular glutamate transporter type 3, glutamate/GABA co-transmission, type III mGluRs

## Abstract

Hippocampal interneurons release the inhibitory transmitter GABA to regulate excitation, rhythm generation and synaptic plasticity. A subpopulation of GABAergic basket cells co-expresses the GABA/glycine vesicular transporters (VIAAT) and the atypical type III vesicular glutamate transporter (VGLUT3); therefore, these cells have the ability to signal with both GABA and glutamate. GABAergic transmission by basket cells has been extensively characterized but nothing is known about the functional implications of VGLUT3-dependent glutamate released by these cells. Here, using VGLUT3-null mice we observed that the loss of VGLUT3 results in a metaplastic shift in synaptic plasticity at Shaeffer’s collaterals – CA1 synapses and an altered theta oscillation. These changes were paralleled by the loss of a VGLUT3-dependent inhibition of GABAergic current in CA1 pyramidal layer. Therefore presynaptic type III metabotropic could be activated by glutamate released from VGLUT3-positive interneurons. This putative presynaptic heterologous feedback mechanism inhibits local GABAergic tone and regulates the hippocampal neuronal network.

## Introduction

The hippocampus is centrally engaged in the regulation of spatial and emotional memory. In the hippocampus, different classes of interneurons interact with specific cellular domains of pyramidal cells to modulate cell firing, plasticity and network activity. These interneurons form a complex group composed of up to 21 types of cells displaying a variety of molecular expression profiles and distinct morphological and physiological features (Somogyi and Klausberger, 2005; Klausberger and Somogyi, 2008). Basket cells are interneurons that form a dense plexus of inhibitory terminals on the soma of pyramidal cells. Two types of basket cells have been identified: the classic parvalbumin-positive (PV) fast-firing interneurons and the regular-firing cholecystokinin-expressing (CCK) interneurons. PV basket interneurons contribute to the generation of oscillations and are considered the “clocks” of the hippocampus (Cobb et al., 1995; Bartos et al., 2007; Korotkova et al., 2010; Amilhon et al., 2015). The functions of CCK interneurons are less well known; however, anatomical and physiological evidence suggests that these interneurons are intensely innervated by neurons from the raphe and respond to cannabinoids (Freund and Katona, 2007). CCK interneurons are thought to be centrally engaged in mood regulation (Freund and Katona, 2007). Interestingly, some of the CCK interneurons express the atypical vesicular glutamate transporter 3 (VGLUT3) (Somogyi et al., 2004), which indicate that these interneurons may release both glutamate and GABA from their terminals contacting pyramidal cells. However, the role of this dual transmission of GABA and glutamate by basket cells remains unknown.

Unlike VGLUT1 and VGLUT2, VGLUT3 is found mostly in neurons that use transmitters other than glutamate, such as acetylcholine (ACh), serotonin (5-HT) or GABA (for review, see El Mestikawy et al., 2011). VGLUT3 facilitates the vesicular accumulation and transmission of ACh and 5-HT via a mechanism known as “vesicular synergy” (Gras et al., 2008; Amilhon et al., 2010) and VGLUT3-bearing terminals endow ACh and 5-HT fibers with the capacity to use glutamate in fast excitatory neurotransmission (Varga et al., 2009; Higley et al., 2011). Furthermore, it was recently established that glutamate released by VGLUT3-positive ACh interneurons negatively regulate dopamine release in the nucleus accumbens through metabotropic glutamate receptors (mGLURs), (Sakae et al., 2015). Overall, the expression of VGLUT3 in ACh- and 5-HT-expressing neurons contributes to both slow and fast neurotransmission and has significant effects on locomotor activity, reward and anxiety.

Little is known about the paradoxical co-transmission of GABA and glutamate and its putative effects on hippocampal network. The main goal of this work was to describe the molecular, cellular, and integrated properties of VGLUT3 in the hippocampus. We observed that VGLUT3 enhances vesicular GABA filling and endows these inhibitory interneurons with the ability to putatively co-release glutamate. In addition, we showed that VGLUT3 deletion alters synaptic plasticity and metaplasticity as well as theta oscillations in the hippocampus. These observations suggest that glutamate released by VGLUT3-expressing basket cells could inhibit GABAergic synaptic transmission by activating presynaptic metabotropic glutamate receptors (mGLURs).

## Materials and Methods

### Animals

Animal care and handling was performed according to the guidelines of the Canadian Council on Animal Care^1^ and approved by the Facility Animal Care Committee of the Douglas Research Center. The VGLUT3^-/-^ mouse line used in the present study was obtained as previously described (Gras et al., 2008; Amilhon et al., 2010) and backcrossed with 10 generations (F10) of mice with a C57BL/6N background (99% homozygous). Heterozygous mice were bred to generate VGLUT3^-/-^ mice and wild type littermates.

The VGLUT3^flox/flox^ mice Slc17a8 mutant mouse line was established at Phenomin - iCS (Phenomin- Institut Clinique de la Souris-, Illkirch, France^2^). In brief, the targeting vector was constructed as follows (**Figure 4A**). A 0.8 kb fragment encompassing exon 2 was amplified by PCR (from C57Bl/6N ES cells genomic DNA) subcloned in a Phenomin-iCS proprietary vector. This Phenomin-iCS vector contains a LoxP site as well as a floxed and flipped Neomycin resistance cassette. A 4.5 kb fragment (corresponding to the 5′ homology arm) and 3.1 kb fragment (corresponding to the 3′ homology arms) were amplified by PCR and subcloned in step1 plasmid to generate the final targeting construct. The linearized construct was electroporated in C57Bl/6N mouse embryonic stem (ES) cells. After selection, targeted clones were identified by PCR using external primers and further confirmed by Southern blot with 5′ and 3′ external probes. Two positive ES clones were injected into BALB/cN blastocysts, and male chimeras derived gave germline transmission. Mice lacking VGLUT3 selectively in GABAergic neurons (VGLUT3^V IAAT-Cre-flox/flox^) were generated by breeding VIAAT^-ires-cre^ mice (The Jackson laboratory, stock No: 016962) with VGLUT3^flox/flox^ mice.

Mice were housed in groups of three to four animals per cage under standard conditions: 22°C and a 12 h light/dark cycle (8:00–20:00 light period) with food and water provided *ad libitum*. All precautions were taken to minimize the number of animals used and their suffering they may have had to undergo.

### Autoradiography of Type I Cannabinoid Receptor (CB1)

CB1 autoradiography was performed as previously described (Herkenham et al., 1991). Briefly, fresh frozen brain sections (10 μm) were incubated for 3 h at RT° in a buffer containing Tris HCl (50 mM, pH7.4), bovine serum albumin (5%) and [^3^H] CP-55 940 (10 nM, NEN, Boston, MA, USA) in the absence or presence of AM251 (10 mM, Tocris Bioscience) to determine total and non-specific binding, respectively. Slides were washed in Tris HCl (50 mM) containing bovine serum albumin (1%, fatty acid-free) for three times (30 min each) at 4°C dipped in ice-cold distilled water. Dried sections were apposed to tritium-sensitive screen (BAS-TR Fuji Imaging) for a period of 7 days and developed with the FujiBAS 7000 PhosphoImager. Densitometry was analyzed with MCID software, using the standard curve generated from [^3^H]-standards. Specific binding measured in each structure was determined by subtracting the non-specific binding image from that of total binding.

### Radioactive *In Situ* Hybridization

*In situ* hybridization was performed as previously reported (Gras et al., 2008; Vigneault et al., 2015). Shortly, sense or antisense oligonucleotides specific for CB1 or for the floxed exon 2 of VGLUT3 (**Tables 1, 2**, generated by Oramacell) were labeled with [^35^S]-dATP (GE Healthcare) using terminal deoxynucleotidyl transferase (GE Healthcare) to a specific activity of 1–3 × 10^8^ dpm/mg. Mouse brain fresh frozen sections (10 μm) were incubated with hybridization medium (Oramacell, Paris, France) containing 1.4 μl of the labeled oligonucleotides, washed, dried and exposed to a BAS-SR Fujifilm Imaging Plate for 5 days. Plates were scanned with a Fujifilm BioImaging Analyser BAS-5000.

**Table 1:** Oligonucleotides used for *in situ* hybridization detection of CB1 receptors.

**Antisense oligonucleotides**	
mouse CB1_AS1	GGTTTCCTCTCTAGGGGACTCTGATCGCAGGACC
mouse CB1_AS2	CTAATACTCCAGCATCCGGAGCCCTCCTCTGTGC
mouse CB1_AS3	CAAACAGAGCCAGGTACTGGGCTGTCACCCTCTG
mouse CB1_AS4	CCACGGCAGAGATGTCATCAGAAGCTAGCCACCC
mouse CB1_AS5	GATTGCAAGAAGGGGTACTGCCCTGTCAGGCTGG
**Sense oligonucleotides**	
mouse CB1_S1	GGTCCTGCGATCAGAGTCCCCTAGAGAGGAAACC
mouse CB1_S2	GCACAGAGGAGGGCTCCGGATGCTGGAGTATTAG
mouse CB1_S3	CAGAGGGTGACAGCCCAGTACCTGGCTCTGTTTG
mouse CB1_S4	GGGTGGCTAGCTTCTGATGACATCTCTGCCGTGG
mouse CB1_S5	CCAGCCTGACAGGGCAGTACCCCTTCTTGCAATC

**Table 2:** Oligonucleotides used for *in situ* hybridization detection of VGLUT3 floxed-exon 2.

VGLUT3-exon 2 AS1	CAGCTGCAGTCACAGATGTACCGCTTGGGGATG
VGLUT3-exon 2 AS2	CTCTGGACGTCTGCACGGGCCTTCCTTCTTCAT

### Double Fluorescence *In Situ* Hybridization (FISH)

Antisense cRNA riboprobes were obtained from the transcription of a PCR template amplified with primers containing the T7 promoter followed by the vesicular inhibitory amino acid transporter (VIAAT) sequence (forward primer 5′-AATTAACCCTCACTAAAGGGAGCCAGGGCCTGCAGATGGAC-3′ and reverse primer 5′-TAATACGACTCACTATAGGGTCGCTGGGCTGCTGCATGTT-3′) or glutamic acid decarboxylase (GAD) sequence (forward primer 5′-AATTAACCCTCACTAAAGGGAGAGGAGCGGATCCTAATACTACC-3′ and reverse primer 5′-TAATACGACTCACTATAGGGAGATCCATGAGAACAAACACGGG-3′) or the VGLUT3 sequence (forward primer 5′-AATTAACCCTCACTAAAGGGAGAAAAACAGGACTGGGCTGACCC-3′ and reverse primer 5′-TAATACGACTCACTATAGGGAGAGAGACCAAGGTCCATATTCCC-3′). GAD and VIAAT or VGLUT3 riboprobes were labeled with UTPs coupled to fluorescein and digoxigenin, respectively (Roche Applied Science, Indianapolis, IN, USA). Cerebral coronal cryosections (10 μm) were fixed with 4% formaldehyde and hybridized as previously described (Gras et al., 2002). Sections were incubated with anti-fluorescein antiserum coupled with horseradish peroxidase (1:250, 1 h RT, Roche Applied Science). The signal was amplified with the TSA-plus-biotin kit (Perkin Elmer, Waltham, MA, USA). GAD or VIAAT RNA was visualized with Neutravidin Oregon Green (Invitrogen) at 488 nm. After the horseradish peroxidase was inactivated with a glycine solution, the brain slices were incubated in the blocking solution for 30 min at RT. The slices were then incubated with anti-DIG coupled with horseradish peroxidase (1:2500, Roche Applied Science) for 1 h at RT. The TSA-plus-Cyanine 3 kit (Perkin Elmer, 10 min at room temperature) was used to detect the VGLUT3 transcript under 555 nm-excitation fluorescence light. The slices were mounted with Fluoromount-G (Southern Biotech, Birmingham, AL, USA) and analyzed with an AxioObserver.Z1 microscope (Carl Zeiss, Germany).

### Immunofluorescent Imaging

Immunofluorescence experiments were performed as previously described (Amilhon et al., 2010). Coronal mouse brain sections that included the ventral hippocampus were incubated with VGLUT3 guinea-pig antiserum [1:3000 (Amilhon et al., 2010)], VIAAT rabbit antiserum (1:3000 (Dumoulin et al., 1999)), gephyrin mouse antiserum (1:1000, Synaptic System, Göttingen, Germany), mGLUR4 rabbit antiserum (1:100, Invitrogen, Life Technologies Inc., Burlington, ON, Canada, cat. # 51-3100), mGLUR7 rabbit antiserum (1:1000, homemade), CB1 rabbit antiserum (1 : 500, generous gift from Zsolt Lenkei, UMR8249, ESPCI-ParisTech. (Thibault et al., 2013)) and DAPI to stain nuclei. Primary antisera were detected with Alexa-fluor-488- or Alexa-fluor-555-conjugated secondary antibodies (1:2000, Molecular Probes Inc., USA). Images were acquired using an AxioObserver.Z1 microscope connected to an Apotome.2 module (Carl Zeiss, Germany). For 3D reconstruction, pictures were captured using a Leica TSC SP2 upright laser scanning confocal microscope (Leica MicroSystems, Germany) equipped with a 63×, 1.4 NA oil immersion objective with the pixel size set to 60 nm and a z-step of 130 nm. Images were deconvoluted using a Maximum Likelihood Estimation algorithm with Huygens 3.6 software (Scientific Volume Imaging). The background intensity was estimated from the average of the voxels with the lowest intensity, and the signal-to-noise ratio was assigned an estimated value of 20. Labeled terminals were quantified using the JaCop plugin of ImageJ (Bolte and Cordelieres, 2006). Co-localization analyses were performed by correlating the intensities of pairs of pixels in the two different channels as previously described (Jaskolski et al., 2005) using the co-localization color map plugin of ImageJ.

### Electron Microscopy

VGLUT3 was detected with an electron microscope with a post-embedding immunogold method as previously described (Gras et al., 2002, 2008). Sagittal brain sections of the hippocampus were incubated with guinea pig anti-VGLUT3 antiserum (1:1000). Immunolabeling was detected with goat anti-guinea pig IgGs conjugated to gold particles (1.4 nm in diameter; Nanoprobes, NY, USA, 1:100). After post-fixation (1% glutaraldehyde), the immunogold signal was enhanced with a silver enhancement kit (HQ silver, Nanoprobes; NY, USA). Ultrathin sections were examined with a transmission electron microscope (EM 912 OMEGA, ZEISS).

### Immuno-Isolation of Synaptic Vesicles, Western Blot, and [^3^H]glutamate Uptake Assay

Synaptosomes from hippocampus were prepared, and the LS1 fractions containing synaptic vesicles (SVs) were isolated as described previously (Zander et al., 2010). The LS1 fractions were incubated with ^∗^superparamagnetic beads (Dynabeads Pan Mouse IgG, Invitrogen, Life Technologies Inc., Burlington, ON, Canada) coupled to anti-synaptophysin, anti-VGLUT1 or anti-VIAAT antisera (all from Synaptic Systems, Göttingen, Germany). Beads without primary antibodies were used as negative controls. Bead-bound SVs were dissolved in Laemmli buffer, and the resultant protein pattern was analyzed by SDS-PAGE/Western blot (Zander et al., 2010) using anti-VGLUT1, anti-VIAAT, anti-synaptophysin and anti-VGLUT3 (all from Synaptic Systems, Göttingen, Germany). Immuno-signals were visualized by enhanced chemiluminescence. Densitometric analyses were performed using LabImage 1D 2006 (Kapelan Bio-Imaging Solutions) and quantified using standard curves obtained from the initial LS1 fraction.

Vesicular [^3^H]glutamate uptake analyses were performed as described previously (Zander et al., 2010; Frahm et al., 2015). LS1-immunoisolated SVs were bound to M-280 sheep anti-mouse beads (Invitrogen). Beads coupled with non-specific mouse IgG (Santa Cruz Biotechnologies) were used as negative controls. Uptake assays were performed with immunoisolated SVs and initiated by the addition of L-glutamate (49.5 μM, Sigma–Aldrich) and [^3^H]-glutamate (0.5 μM, Hartmann Analytic GmbH, Germany). Uptake was stopped after 10 min at 25°C, and unbound radioactivity was removed by centrifugation (435,000 × *g*, 10 min).

### Patch-Clamp and Field Potential Recordings on Hippocampal Slices

Mice (23–35 days old) were anesthetized with isoflurane and decapitated. Their brains were quickly removed and placed in an oxygenated (95% O2/5% CO_2_ gas mixture) icy slicing solution containing (in mM): 2.5 KCl, 0.1 CaCl_2_, 4 MgCl_2_, 1.25 KH_2_PO_4_, 25.2 sucrose, 26 NaHCO_3_ and 10 glucose (Manseau et al., 2010). Coronal slices (300 μm) containing the ventral hippocampus were cut with a vibratome (vt1200, Leica Microsystems Inc., Canada) and placed in a slice saver filled with oxygenated artificial cerebrospinal fluid [ACSF, 125 mM NaCl, 2.5 mM KCl, 2 mM CaCl_2_, 2 mM MgCl_2_, 1.25 mM NaH_2_PO_4_, 26 mM NaHCO_3_ and 25 mM glucose (Manseau et al., 2010)]. The slices were incubated for at least 1 h at RT prior to experimentation.

For patch-clamp experiments, evoked and spontaneous miniature inhibitory postsynaptic potentials (eIPSCs and mIPSCs, respectively) were recorded in CA1 pyramidal neurons. Recordings were performed using the whole cell configuration of the patch-clamp technique with borosilicate glass electrodes (tip resistance: 2.5–3.5 MΩ) filled with a “high chloride” intracellular solution (70 mM *K*-gluconate, 70 mM KCl, 2 mM NaCl, 2 mM MgCl_2_, 10 mM HEPES, 1 mM EGTA, 5 mM QX-314, 2 mM Mg-ATP, and 0.3 mM Na-GTP (Manseau et al., 2010)). The *E*_Cl_ estimated using the Nernst equation was approximately -16 mV. Action potentials were blocked with QX-314 (5 mM). Under these conditions, monosynaptically elicited eIPSCs in a pyramidal neuron held at -70 mV were represented by an inward current. Fast glutamatergic transmissions were blocked with DNQX and AP5 for all patch-clamp recordings. The perfusion of bicuculline (5 μM) completely abolished the recorded inward current, which confirmed the GABAergic nature of the postsynaptic events (see Supplementary Figure S1). An inhibitory square pulse was applied before each stimulation to measure the input resistance and assess the quality of the patch. Recordings were excluded if the input resistance varied more than 20%. Although the theoretical ECl^-^ was estimated to be approximately -16 mV by the Nernst equation, the measured ECl^-^ was approximately -8 mV on the IV curve of GABAergic currents (*n* = 5, see Supplementary Figure S1). The membrane potential was clamped at -70 and -80 mV for eIPSC and mIPSC recordings, respectively. Spontaneous mIPSCs were recorded in the presence of tetrodotoxin (TTX, 0.5 μM). The eIPSCs were evoked monosynaptically by placing a borosilicate glass pipette in the stratum pyramidale 50 μm away from the recorded neuron. The signals were amplified with a Multiclamp 700B patch-clamp amplifier (Axon Instruments, Foster City, CA, USA), sampled at 20 kHz and filtered at 10 kHz using a Multiclamp 700B commander (Axon Instruments). The data were analyzed using pClamp (Axon Instruments) and Origin (Microcal Software, Northampton, MA, USA) software. A custom-written software was used for the mIPSC analysis (Manseau et al., 2010).

For long term potentiation/depression (LTP/LTD) recordings, field excitatory postsynaptic potentials (fEPSPs) were recorded in the stratum radiatum of the medial CA1 region with a borosilicate glass electrode (tip resistance: 2.7–3.3 MΩ) filled with ACSF at RT. The fEPSPs were evoked every 20 s with a 80 μsec stimulation at afferent Schaffer collateral fibers delivered through a stimulus isolator (WPI) with a bipolar tungsten-based electrode. The stimulation intensity was adjusted to obtain amplitudes close to 50% of the maximum response. The fEPSP baseline was monitored for 20 min. The synaptic transmission was potentiated either by a high-frequency tetanic stimulation (HFS) consisting of three trains of 100 pulses 20 s apart at a frequency of 100 Hz or a theta-burst stimulation (TBS) consisting of 3 trains 20 s apart of 10 bursts of 5 pulses at 100 Hz at a burst frequency of 5 Hz. The fEPSP slope is defined as the slope of the linear fit of the portion of the field curve between 10 and 60% of maximum peak amplitude. The synaptic transmission was depressed by a low-frequency stimulation consisting of 900 pulses at either 1 Hz (LFS-1 Hz) or 3 Hz frequency (LFS-3 Hz). When stated, bicuculline (5 μM) was added to the ASCF throughout the experiment to block GABA_A_ receptor-mediated fast inhibitory transmission. Long-term potentation (LTP) or long-term depression (LTD) are defined as the comparison of the mean fEPSP slope over the last 10 min of recording after stimulation (30–40 min. for LTP, 50–60 min. for LTD) with the mean fEPSP slope over the 10 min prior to stimulation.

### Oscillation Recordings in the Entire Hippocampus Preparation *In Vitro*

Intact hippocampi were isolated from 21- to 25-day-old mice as previously described (Khalilov et al., 1997; Tretter et al., 2012; Amilhon et al., 2015). Briefly, the hippocampi were dissected and placed in an oxygenated high-sucrose solution before use. Recordings were made with 4.0 mM KCl ACSF filled borosilicate pipette (Goutagny et al., 2009). Local field potentials (LFP) recorded in the CA1 stratum radiatum of the temporal part of the hippocampus were filtered online (0.1–500 Hz) and sampled at 5 kHz. Rhythmic oscillations ranging from 3 to 12 Hz were considered to represent theta oscillations.

### Chemicals

With the exception of bicuculline (Sigma–Aldrich) and tetrodotoxine (TTX, Alomone, Israel), all drugs were purchased from Ascent Scientific (Abcam Biochemicals), including D-(-)-2-Amino-5-phosphonopentanoic acid (AP5), 6,7-Dinitroquinoxaline-2,3-dione (DNQX), (2*S*)-2-Amino-2-[(1*S*,2*S*)-2-carboxycycloprop-1-yl]-3-(xanth-9-yl)propanoic acid (LY341495), (4-Methoxyphenyl)-(6-methoxy-quinazolin-4-yl)amine (LY456236), 2-Methyl-6-(phenylethynyl) pyridine (MPEP) and (*RS*)-α-Methylserine-*O*-phosphate (MSOP) and WIN55,212-2 mesylate.

### Statistics

Statistical analyses were performed using Prism 4 software (Graph Pad). Each statistical test was appropriately chosen for the relevant experimental design. To compare two groups, a two-tailed paired Student *t*-test or a non-parametric Mann–Whitney test was performed according to normality of the group distributions. For multiple group comparisons, a one- or two-way ANOVA or repeated measure ANOVA was performed followed by a *post hoc* Bonferroni comparison of the group means. Data are expressed mean ± SEM. ^∗^*P* < 0.05, ^∗∗^*P* < 0.01, ^∗∗∗^*P* < 0.001. A descriptive table of all the statistics is provided in the Supplementary Tables S1–S5.

## Results

### VGLUT3 Is Expressed by a Subclass of GABA Terminals in CA1

**FIGURE 1:**
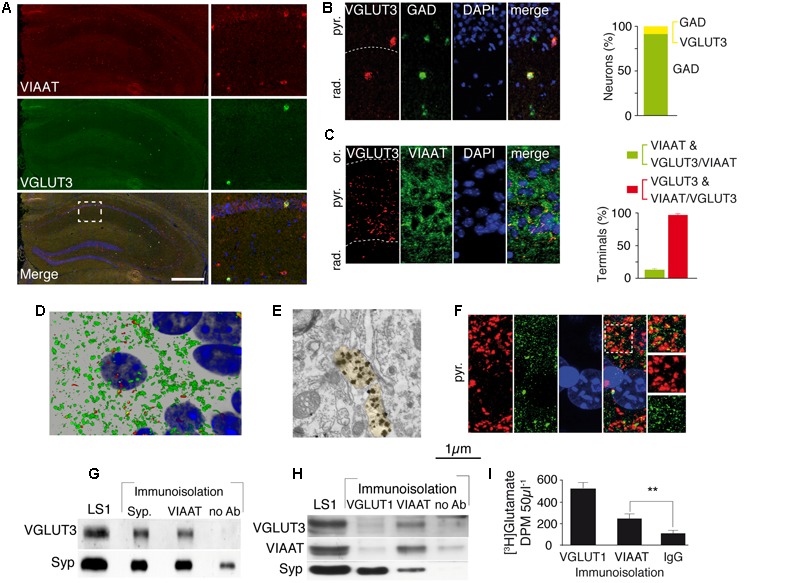
**VGLUT3 is present in the GABAergic terminals and synaptic vesicles of basket cells of the CA1 layer. (A)** Double FISH labeling of mRNA encoding VIAAT (red) and VGLUT3 (green) on a coronal section from WT mouse hippocampus. Nuclei were stained with DAPI (blue). An enlargement of the CA1 area is shown on right panels. **(B)** Double FISH labeling of mRNA encoding VGLUT3 (red) and GAD (green) in CA1 pyramidal cell layer. Nuclei are stained with DAPI (blue). Quantification of neurons synthesizing GAD (green bar) or VGLUT3 and GAD (yellow bar) **(C)** Immunofluorescence labeling of VGLUT3 (red), VIAAT (green), and DAPI (blue). Note the dense VGLUT3 labeling in the pyramidal cell layer (delineated by a dashed line). Histogram represents the quantification of VGLUT3 and VIAAT co-localization in GABAergic terminals from CA1 pyramidal layer. **(D)** 3D reconstruction of VIAAT (green)- and/or VGLUT3 (red)-positive terminals surrounding a pyramidal cell detected by its nucleus (blue). **(E)** VGLUT3-positive gold immunoparticles detected by electron microscopy in 2 side-by-side axo-somatic terminals (light yellow) making symmetrical synapses. **(F)** Frequent apposition of VGLUT3- (red) and gephyrin-(green) positive terminals. **(G)** Immunoisolation of synaptic vesicles from hippocampal LS1 fractions. Immunoisolated VIAAT-positive vesicles co-express VGLUT3. Synaptophysin-containing vesicles express both synaptophysin and VGLUT3 (positive control). No VGLUT3 vesicles were immunoisolated in the absence of antibodies (negative control). **(H)** Immunoisolated VIAAT-containing vesicles co-express VGLUT3 and synaptophysin, whereas vesicles immunoisolated with VGLUT1 co-express synaptophysin but not VIAAT or VGLUT3, which demonstrates the specificity of the immunoisolation technique. **(I)** Glutamate uptake measured in immunoisolated glutamatergic (VGLUT1-positive) or GABAergic (VIAAT-positive) vesicles. VIAAT-isolated vesicles take up glutamate to a lesser extent than pure glutamatergic vesicles but to a significantly greater extent than the negative control (IgG). Scale bars in **(A)**: 500 μm in A (insets: 150 μm); 40 μm in **(B)** and **(F)**; 30 μm in **(C)**, 10 μm in **(D)**; 0.250 μm in **(E)**. ^∗∗^*P* < 0.01.

In the hippocampus, VGLUT3 is expressed by CCK-positive and more rarely by calbindin-positive interneurons (Somogyi et al., 2004; Klausberger and Somogyi, 2008). We first determined the percentage of GABAergic interneurons expressing VGLUT3. A double FISH technique was used to label the mRNA encoding VGLUT3 and VIAAT (**Figure 1A**) or glutamic acid decarboxylase (GAD, the GABA-synthesizing enzyme, **Figure 1B**). We found that 8.7 ± 0.3% of all GAD expressing GABAergic neurons in the hippocampus expressed VGLUT3 (**Figure 1B**). Similar results were obtained with VIAAT-expressing GABAergic neurons (not shown). Once synthesized, VGLUT3 is transported to the axon terminals surrounding the somata of neurons in the pyramidal layer of the hippocampus (**Figures 1C,D**). We determined by three-dimensional non-biased counting that 98 ± 1% of VGLUT3-positive terminals co-expressed VIAAT but only 13 ± 2% of VIAAT-positive terminals contained VGLUT3 in the CA1 pyramidal layer of the hippocampus. Electron microscopy showed that VGLUT3-immunoglod puncta were present over synaptic vesicle clusters in axon terminals forming axo-somatic symmetrical synapses (**Figure 1E**). VGLUT3-positive terminals co-localized with gephyrin (**Figure 1F**), a major scaffolding protein found at inhibitory synapses (Freund and Buzsaki, 1996). These results demonstrated that only a minority of functional GABAergic synapses in the pyramidal cell layer expressed VGLUT3.

We next assessed whether VGLUT3 and VIAAT were co-localized within the same SVs. After immunoisolation of VGLUT1- or VIAAT-containing SVs, VGLUT3 was recovered only from VIAAT-isolated SVs (**Figures 1G,H**). Moreover, VIAAT-positive SVs were able to accumulate [^3^H]glutamate (VIAAT, *n* = 4; IgG, *n* = 4, Student *t*-test, unpaired, *P* = 0.0028, *t* = 2.65, **Figure 1I**). These results demonstrated that VGLUT3 was co-expressed with VIAAT in SVs and that these SVs could readily concentrate glutamate, and might have the ability to release both glutamate and GABA.

### VGLUT3 Ablation Modified the Network Activity and the Plasticity of the Hippocampus

GABAergic inputs inhibit individual pyramidal cells and synchronize large groups of excitatory neurons. PV-positive basket cells may play an important role in generating hippocampal rhythms, and CCK-positive basket cells may modulate network activity in a behaviorally and environmentally contingent manner (Bliss and Collingridge, 1993; Klausberger and Somogyi, 2008; Lapray et al., 2012). We examined whether the absence of VGLUT3 significantly affected theta rhythms and long-term synaptic plasticity, two important phenomena involved in learning, memory and emotional processing.

**FIGURE 2:**
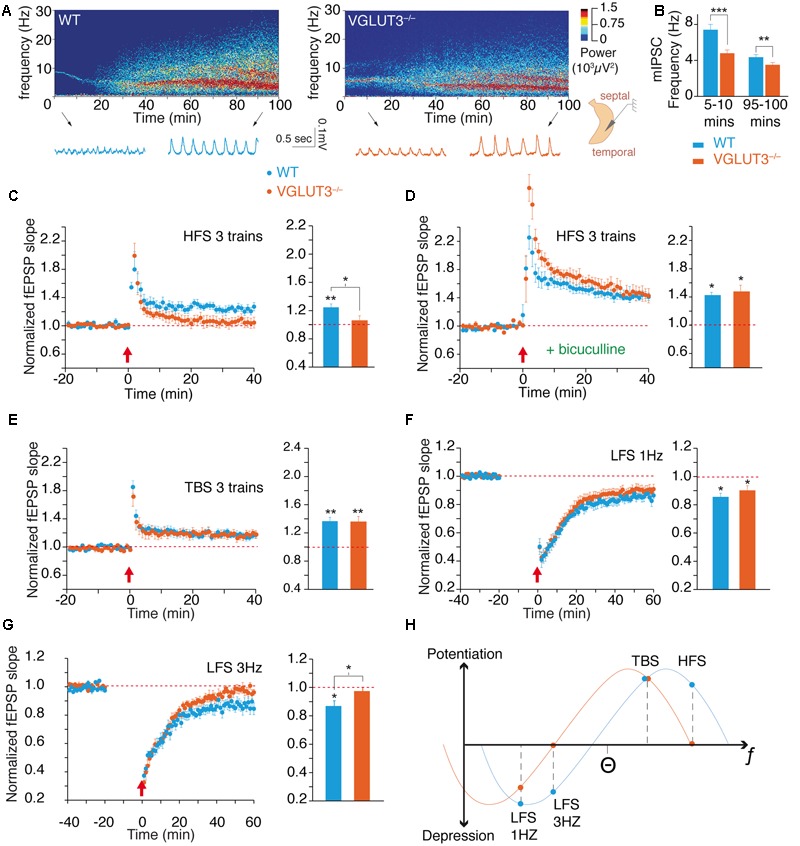
**VGLUT3 regulates the theta oscillations and the metaplasticity of hippocampus network. (A)** Examples of theta oscillation recordings in the CA1 region of an intact hippocampus preparation *in vitro*. The upper panels represent power spectrograms over time (power is coded in color) in the WT and VGLUT3^-/-^ (*n* = 12–14) mice. Oscillations are shown under the power spectrograms at the beginning (5–10 min) and end (95–100 min) of the recordings. **(B)** Mean frequency of theta oscillations at the beginning and end of the recordings. Theta frequency decreased over time and was significantly slower in the VGLUT3^-/-^ mice than in the WT mice (*n* = 14–15). **(C)** LTP at Schaffer collateral-CA1 (Sch-CA1) synapses induced by HFS (3 × 100 Hz, red arrow) in WT and VGLUT3^-/-^ (*n* = 9–10) mice. Histogram showing the mean value of the fEPSP slope 40 min after LTP induction. Significant LTP was induced in the WT mice, whereas no significant LTP was induced in the VGLUT3^-/-^ mice (*n* = 9–10). **(D)** LTP at Sch-CA1 synapses induced by HFS (red arrow) after GABA-A receptor blockade with bicuculline (5 μM). Histogram showing the mean value of the fEPSP slope 40 min after LTP induction. A LTP of similar amplitude was induced in the WT and VGLUT3^-/-^ mice (*n* = 6). **(E)** LTP at Sch-CA1 synapses induced by TBS (red arrow). Histogram showing the mean value of the fEPSP slope 40 min after LTP induction. A LTP of similar amplitude was induced in the WT and VGLUT3^-/-^ mice (*n* = 8–9). **(F)** LTD at Sch-CA1 synapses induced by LFS (1 Hz, 15 min, red arrow). Histogram showing the mean value of the fEPSP slope 60 min after LTD induction. A LTD of similar amplitude was induced in the WT and VGLUT3^-/-^ mice (*n* = 6–9). **(G)** LTD at Sch-CA1 synapses induced by LFS (3 Hz, 15 min., red arrow) in WT and VGLUT3^-/-^ mice. Histogram showing the mean value of the fEPSP slope 60 min after LTD induction. Significant LTD was induced in the WT mice (*n* = 7), whereas no significant LTD was induced in the VGLUT3^-/-^ mice (*n* = 7). **(H)** Schematic representation of the plot of plasticity amplitude as a function of the strength of the stimulation protocol in the presence or absence of VGLUT3. The curve obtained with VGLUT3^-/-^ mice is shifted toward lower frequencies. This indicates that VGLUT3^-/-^ mice are still capable of bidirectional plasticity in the hippocampus. However, the loss of VGLUT3 strongly increases local GABA transmission and hence slows down the entire network and shifts metaplasticity toward the left. ^∗^*P* < 0.05, ^∗∗^*P* < 0.01, ^∗∗∗^*P* < 0.001.

Theta oscillations were recorded in the CA1 area of the ventral hippocampus from a complete preparation *in vitro* (**Figures 2A,B**) (Khalilov et al., 1997; Amilhon et al., 2015). These theta oscillations are generated intrinsically in the hippocampus by the delicate interactions between interneurons and pyramidal cells (Khalilov et al., 1997; Amilhon et al., 2015). The frequency of theta oscillations was significantly lower in the VGLUT3^-/-^ mice than in WT littermates throughout recordings during the first 5–10 min [WT (*n* = 12): 7.4 ± 0.6 Hz and VGLUT3^-/-^ (*n* = 14): 4.8 ± 0.4 Hz, Mann–Whitney test, *P* = 0.0002, *U* = 9.5] or recordings during the last 95–100 min (WT: 4.4 ± 0.3 Hz and VGLUT3^-/-^: 3.5 ± 0.3 Hz, Mann–Whitney test, *P* = 0.0054, *U* = 29.5, **Figure 2B**). However, no significant differences in theta power between groups were observed (not shown). Therefore, the absence of VGLUT3 impaired the excitatory-inhibitory balance required for normal oscillatory rhythmic activity in the hippocampus.

Long-term plasticity of synaptic transmission is a molecular and cellular process that underlies learning and memory through the fine-tuning of synaptic strength (Cooke and Bliss, 2006; Omiya et al., 2015). Using CA1 field potential recordings and extracellular stimulations we examined whether VGLUT3 loss modulates the expression of LTP and LTD. Under conditions of normal glutamate and GABA transmission, tetanic high-frequency stimulation (HFS; see Materials and Methods) of Schaffer’s collateral-CA1 connections led to a long-term potentiation of +24 ± 5% of field EPSP (fEPSP) slope in WT mice (*n* = 9, Wilcoxon test, *P* = 0.0039, *W* = -45, **Figure 2C**). In VGLUT3^-/-^ mice no LTP was significantly induced by HFS stimulation (+5 ± 7%, *n* = 10, Wilcoxon test, *P* = 0.4922, *W* = -15, **Figure 2C**). Therefore, the absence of VGLUT3 resulted in a significant difference in HFS-induced LTP between genotypes (Mann–Whitney test, *P* = 0.0350, *U* = 19).

When fast GABAergic transmission was suppressed with application of the GABA_A_ receptor antagonist bicuculline (5 μM), in WT mice HFS-induced LTP was of +41 ± 2% (*n* = 6, Wilcoxon test, *P =* 0.0313, *W* = -21, **Figure 2D**) and in VGLUT3^-/-^ mice was of: +45 ± 10% (*n* = 6, Wilcoxon test, *P* = 0.0313, *W* = -21, **Figure 2D**). Hence, in the absence of GABAergic transmission, HFS-LTPs were of comparable amplitude in WT and VGLUT3 null mice (Mann–Whitney test, *P* = 0.3095, *U* = 11).

Theta burst stimulation (TBS; see Materials and Methods) is a pattern of activity of milder intensity but considered more physiologically relevant than tetanic HFS. We wondered whether the loss of VGLUT3 would also affect TBS-induced LTP. TBS induced a significant LTP in WT mice of +16,6 ± 5% (*n* = 9, Wilcoxon test, *P* = 0.0039, *W* = -49, **Figure 2E**) as well as in VGLUT3^-/-^ mice: +16,8 ± 4% (*n* = 8, Wilcoxon test, *P* = 0.0078, *W* = -36, **Figure 2E**). The LTP elicited in both groups were very similar in amplitude (Mann–Whitney test, *P* > 0.99, *U* = 36). These results suggest that the loss of VGLUT3 affected specific patterns of activity rather than blunting synaptic plasticity in general.

To further investigate this hypothesis, we investigated how VGLUT3 deletion altered LTD. LTD is generally induced by low frequency patterns of inputs between 0.5 and 10 Hz. The application of a low-frequency stimulation of 900 pulses at 1 Hz (LFS-1 Hz) led to a synaptic depression in WT group of -14,4 ± 2,6% (*n* = 6, Wilcoxon test, *P* = 0.0313, *W* = 21, **Figure 2F**). It also induced a synaptic depression in VGLUT3^-/-^ mice: -9,8 ± 3,3% (*n* = 9, Wilcoxon test, *P* = 0.0195, *W* = 39, **Figure 2F**). LTDs were comparable in amplitude between WT mice and VGLUT3^-/-^ mice (Mann–Whitney test, *P* = 0.3153, *U* = 18). We then applied a low-frequency stimulation consisting of 900 pulses at a 3 Hz frequency (LFS-3 Hz). After LFS-3 Hz stimulation, a significant depression of -13,1 ± 3,6% was observed in WT mice (*n* = 7, Wilcoxon test, *P* = 0.0313, *W* = 26, **Figure 2G**). However, LFS-3 Hz application failed to induce significant LTD in the VGLUT3^-/-^ mice: -2,6 ± 2,6% (*n* = 7, Wilcoxon test, *P* = 0.2969, *W* = 14, **Figure 2G**). The difference between LTD levels was statistically significant at 3 Hz (Mann–Whitney test, *P* = 0.0379, *U* = 8).

The theoretical curve shown on **Figure 2H** summarizes the long-term plasticity results. Levels of LTP and LTD were plotted in regard to the intensity of the stimulation protocol. These results suggest that the removal of VGLUT3 induces a metaplastic shift of synaptic plasticity toward lower frequencies of induction. Taken together, these results demonstrate the impact of VGLUT3 on the excitatory-inhibitory balance and demonstrate that VGLUT3 is an important player in the regulation of theta oscillations and synaptic plasticity.

### Role of VGLUT3 in GABAergic Transmission

We next investigated how VGLUT3 regulated the excitatory-inhibitory balance of the hippocampal network. In CCK-basket cells, VGLUT3-dependent glutamate could act directly as a neurotransmitter of excitatory synaptic transmission and/or indirectly as a neuromodulator of the inhibitory synaptic transmission.

**FIGURE 3:**
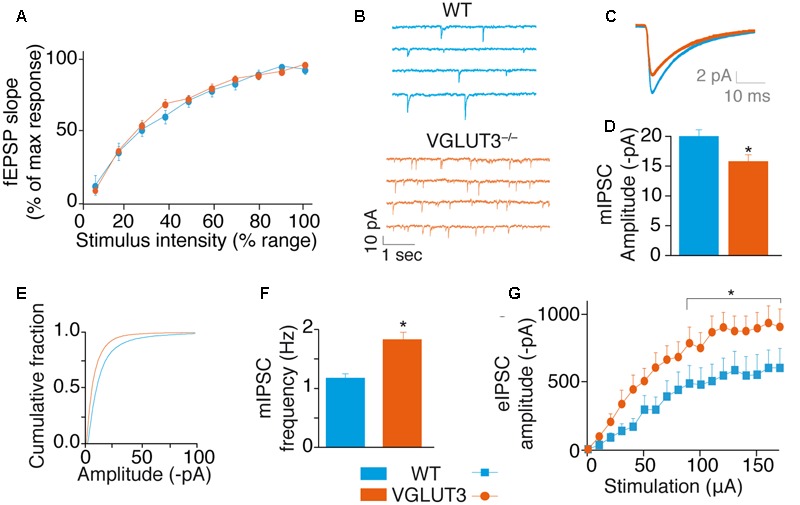
**VGLUT3 regulates GABAergic synaptic transmission. (A)** Input-output curve of fEPSPs at Sch-CA1 synapses showing that the absence of VGLUT3 does not modify fast glutamatergic synaptic transmission in the CA1 region of the hippocampus (WT *n* = 10, VGLUT3^-/-^ *n* = 10). **(B)** Examples mIPSC traces from patch-clamp recordings in CA1 pyramidal neurons of WT mice (*n* = 27) and VGLUT3^-/-^ mice (*n* = 25). Neurons were held at –80 mV. Action potentials were blocked with TTX (0.5 μM) and ionotropic glutamatergic receptors were blocked with DNQX (20 μM) and AP5 (25 μM). **(C)** Averaged mIPSCs in neurons of WT mice (*n* = 27) and VGLUT3^-/-^ mice (*n* = 25). **(D)** Mean amplitude of mIPSCs in neurons of WT mice and VGLUT3^-/-^ mice. **(E)** Cumulative distribution of mIPSCs showing a drift to the left of the curve recorded in the VGLUT3^-/-^ mice. **(F)** Mean mIPSC frequency (WT *n* = 16, VGLUT3^-/-^ *n* = 14). **(G)** Input–output curve showing that evoked GABAergic synaptic transmission increased in the absence of VGLUT3. Monosynaptic eIPSCs were recorded in CA1 pyramidal neurons. Neurons were held at –70 mV and ionotropic glutamatergic receptors were blocked with DNQX (20 μM) and AP5 (25 μM). The plateau reached by the eIPSCs was significantly lower in the WT mice (*n* = 16) than in the VGLUT3^-/-^ mice (*n* = 14). ^∗^*P* < 0.05.

To test VGLUT3-dependent glutamate activity on basal glutamatergic synaptic transmission, we recorded fEPSPs at various stimulation intensities. We built up an input/output curve of fEPSP slopes obtained in the CA1 region of WT and VGLUT3^-/-^ mice (**Figure 3A**). The average maximal amplitude of fEPSPs obtained in each genotype was similar (Mann–Whitney test, *P* = 0,6182, *U* = 43, not shown). When normalized to the maximal amplitude of each slice, the mean slope recorded at each intensity of stimulation was also similar between WT and VGLUT3^-/-^ mouse groups [RM-ANOVA, *P* = 0.6765, *F*(1,18) = 0,1799, **Figure 3A**]. This result suggests that VGLUT3-dependent glutamate did not directly regulate fast glutamatergic transmission.

We then investigated whether VGLUT3-dependent glutamate was a neuromodulator of GABAergic transmission in CA1 pyramidal neurons (**Figures 3B–G**). First we measured mIPSCs under the blockade of action potentials with TTX and of fast synaptic AMPA/Kainate and NMDA glutamate receptors with DNQX (20 μM) and AP-5 (25 μM), respectively. In VGLUT3^-/-^ mice, the mean mIPSCs amplitude was reduced by 27 ± 7% (WT: -20.2 ± 1.4 pA, *n* = 27 and VGLUT3^-/-^: -15.8 ± 1.4 pA, *n* = 25, Mann–Whitney test, *P* = 0.0200, *U* = 210, **Figures 3B–E**). This observation suggested that a vesicular synergy between glutamate and GABA loading in SVs was lost in the absence of VGLUT3 (El Mestikawy et al., 2011).

In addition, the frequency of mIPSCs was increased by 32 ± 7.6% in the VGLUT3^-/-^ mice (1.8 ± 0.2 Hz in VGLUT3^-/-^ compared to 1.2 ± 0.1 Hz in WT littermates, Mann–Whitney test, *P* = 0.0438, *U* = 217.5, **Figure 3F**). These results indicate that VGLUT3 regulates both amplitude and frequency of GABA mIPSCs.

To further understand the changes observed in GABAergic synaptic transmission, we quantified electrically evoked IPSCs (eIPSCs) in WT and VGLUT3^-/-^ mice. Evoked IPSCs were elicited monosynaptically by the direct activation of GABAergic somata and terminals while the fast synaptic AMPA/Kainate and NMDA glutamate receptors were blocked with DNQX (20 μM) and AP-5 (25 μM), respectively (Supplementary Figure S1). The input-output curve of GABA eIPSCs in CA1 pyramidal cells showed that the maximal eIPSC amplitude in pyramidal neurons was significantly increased in the VGLUT3^-/-^ mice (+68 ± 24%, *n* = 14, relative to WT mice, *n* = 16, Mann–Whitney test, *P* = 0.0151, *U* = 63, **Figure 3G**).

These results demonstrate that VGLUT3-dependent glutamate acts as a neuromodulator that strongly inhibits GABAergic transmission.

### VGLUT3-Dependent Glutamate Modulating GABA Transmission Originates from GABAergic Basket Cells

**FIGURE 4:**
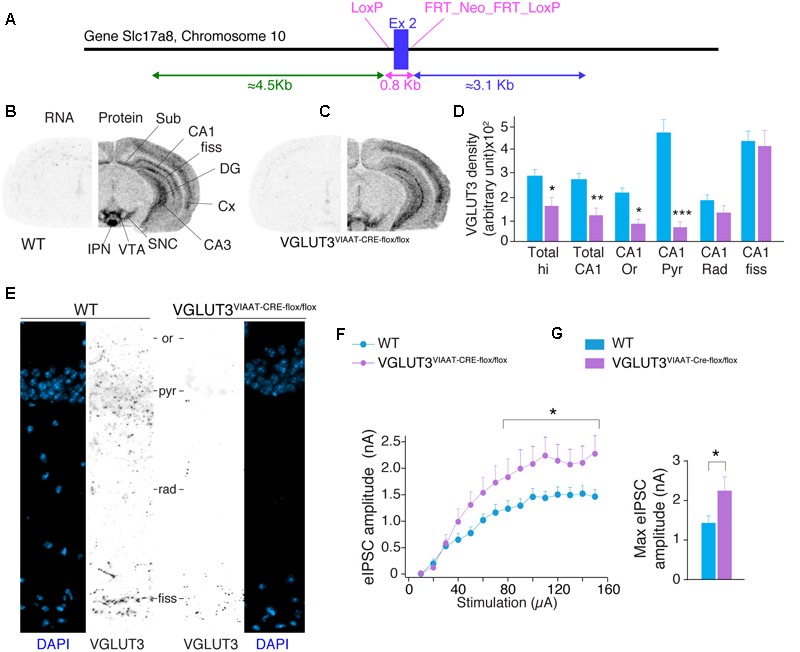
**VGLUT3-dependent glutamate that regulates GABAergic synaptic transmission originates from GABAergic hippocampal interneurons. (A)** Targeting strategy of the *vglut3* gene locus to obtain VGLUT3^flox/flox^ mouse line. LoxP sites flank exon 2. FRT sites flank the positive selectable marker, TK-*neomycin*. VGLUT3^flox/flox^ mice were bred with VIAAT-CRE mice. VGLUT3 expression in WT mice **(B)** and VGLUT3^V IAAT-Cre-flox/flox^ mice **(C)**. VGLUT3 mRNA detected by *in situ* hybridization is shown on the left coronal hemi-section whereas, the protein detected by immunoautoradiography is shown on the right hemi-section. Note the selective ablation of VGLUT3 mRNA in cortical and hippocampal interneurons from VGLUT3^V IAAT-Cre-flox/flox^ mice. **(D)** Quantification of VGLUT3 protein density in the hippocampus, CA1 and its subregions in WT (*n* = 5) and VGLUT3^V IAAT-Cre-flox/flox^ (*n* = 5) mice. **(E)** VGLUT3 immunofluorescence (in black) and nuclei stain with DAPI (blue) in the CA1 region of the hippocampus in WT mice (left panels) and VGLUT3^V IAAT-Cre-flox/flox^ mice (right panels). **(F)** Input–output curve showing that evoked GABAergic synaptic transmission increased when VGLUT3 expression is selectively suppressed in GABAergic interneurons. **(G)** Histogram showing that the averaged maximal eIPSC obtained at the plateau phase of the curve is significantly increased in VGLUT3^V IAAT-Cre-flox/flox^ mice (*n* = 10) compared with their WT littermates (*n* = 10). ^∗^*P* < 0.05, ^∗∗^*P* < 0.01, ^∗∗∗^*P* < 0.001. Abbreviations: Cx, cerebral cortex; DG, dentate gyrus; fiss, hippocampal fissure; IPN, interpeduncular nuclei; Or, *stratum* oriens; Pyr, *stratum* pyramidale; Rad, stratum radiata; SNC, substantia nigra pars compacta; Sub, subiculum; VTA, ventral tegmental area.

To confirm the GABAergic origin of this VGLUT3-dependent regulatory mechanism, we developed mutant mice selectively lacking VGLUT3 in GABAergic neurons. The exon 2 of Slc17a8, the gene coding for VGLUT3, was flanked by LoxP sites (**Figure 4A**). VGLUT3^flox/flox^ mice were bred with VIAAT-CRE mice. *In situ* hybridization was performed with antisense oligonucleotides targeting exon 2 of VGLUT3 in the hippocampus of WT and VGLUT3^V IAAT-Cre-flox/flox^ mice (**Figures 4B,C**, left hemisphere). These experiments showed that VGLUT3 exon 2 was absent from the hippocampus of VGLUT3^V IAAT-Cre-flox/flox^ mice.

VGLUT3 protein was then detected by immunoautoradiography. We found a significant decrease of VGLUT3 expression [TWO-WAY ANOVA, *P* < 0.0001, *F*(5,48) = 68.15] in VGLUT3^V IAAT-Cre-flox/flox^ mice. In these mutant animals, the loss of VGLUT3 in the entire hippocampus was 42 ± 10% (Bonferroni post-test, *p* < 0.05, *t* = 2.792, **Figure 4D**) and 54 ± 7% for the CA1 region (Bonferroni post-test, *p* < 0.01, *t* = 3.363). The analysis of CA1 layers showed specific decreases of VGLUT3 content in the pyramidal layer (-84 ± 3%, Bonferroni post-test, *p* < 0.001, *t* = 9.197), the stratum oriens (-59 ± 6%, Bonferroni post-test, *t* = 2.914, *p* < 0.05) but not in the stratum radiatum (-32 ± 13%, Bonferroni post-test, *p* > 0.05, *t* = 1.456) nor the hippocampal fissure (-5 ± 15%, *p* = Bonferroni post-test, *p* > 0.05, *t* = 0.499). These results were further confirmed at higher magnification with immunofluorescent detection of VGLUT3 in the hippocampus of WT mice and VGLUT3^V IAAT-Cre-flox/flox^ mice (**Figure 4E**).

Input-output curves of eIPSCs were then done to compare the GABAergic synaptic transmission in VGLUT3^V IAAT-Cre-flox/flox^ mice and in WT littermates. The maximal amplitude of eIPSCs was significantly increased by 55.2 ± 22.7% in the VGLUT3^V IAAT-Cre-flox/flox^ mice (Mann–Whitney test, *P* < 0.0028, *U* = 21, *n* = 10, **Figures 4E,F**).

This result demonstrates that VGLUT3-dependent glutamate release by GABAergic basket cells is a potent inhibitory modulator of GABAergic transmission.

### VGLUT3-Dependent Glutamate Released by Basket Cells Operates through mGLUR

**FIGURE 5:**
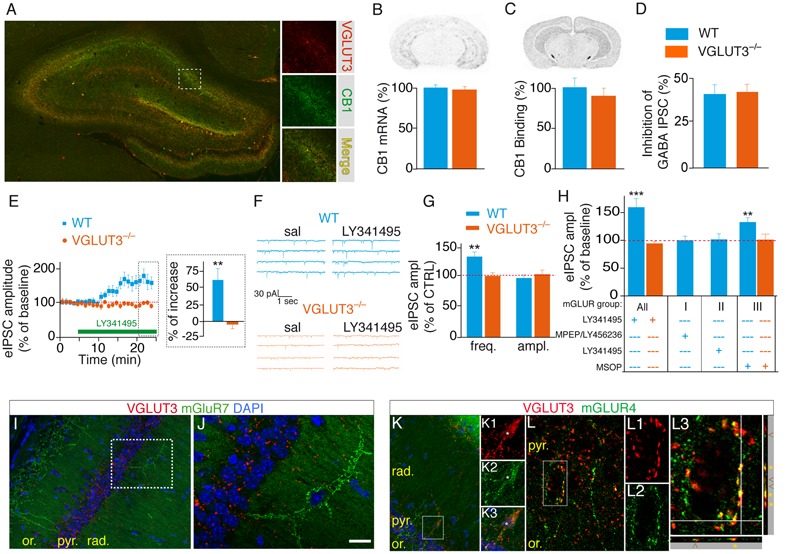
**VGLUT3-dependent regulation of GABAergic synaptic transmission is controlled by type-III mGLURs. (A)** Immunofluorescence labeling of VGLUT3 (red) and CB1 receptors (green) in a WT mouse hippocampus. **(B)** Detection of CB1-mRNA-expressing neurons by *in situ* hybridization. Quantification showed that the number of CB1-positive neurons was similar in the CA1 area of WT and VGLUT3^-/-^ mice (*n* = 5 for each genotype). **(C)** Autoradiographic detection of CB1 receptor labeled with [^3^H]CP-55 940, a tritiated CB1R agonist. The density of CB1 binding sites was similar in the hippocampus of WT and VGLUT3^-/-^ mice (*n* = 5 for each genotype). **(D)** Inhibition of GABAergic eIPSCs by WIN55,212-2 mesylate (a high affinity CB1 agonist) in the CA1 layer of WT mice and VGLUT3^-/-^ mice (*n* = 8 for each genotype). **(E)** The broad-spectrum mGLUR antagonist LY341495 (75 μM) increased the amplitude of eIPSCs in the WT mice but not in the VGLUT3^-/-^ mice (*n* = 11 for each genotype). **(F)** Example mIPSC recordings before (sal, saline) and after LY341495 perfusion. **(G)** LY341495 increased the frequency of mIPSCs in the WT mice but not in the VGLUT3^-/-^ mice (*n* = 9–11). These data also demonstrate the pre-synaptic localization of mGLURs. LY341495 had no effect on the amplitude of mIPSCs in either genotype. **(H)** Effects of broad-spectrum and type I (MPEP, 10 μM plus LY456236, 0.5 μM), II (LY143495, 5 nM) and III (MSOP, 300 μM) mGLUR antagonists on the amplitude of eISPSCs. Types I and II antagonists had no effects on the amplitude of eIPSCs. The blockade of type III mGLURs by MSOP (300 μm) increased the amplitude of eIPSCs to the same extent as L341495 (75 μm) in the WT mice but exerted no effect in the VGLUT3^-/-^ mice (*n* = 6–10). **(I,J)** Immunofluorescence labeling shows the absence of co-expression of VGLUT3 (red) and mGLUR7 (green) in interneurons. **(K,L)** In contrast VGLUT3 and mGLUR4 are found in the soma (K1-3) and terminals (**K**, L1-3) of interneurons. Orthogonal projections show that VGLUT3 and mGLUR4 overlap (yellow stars). Green and red arrowheads indicate distinct neighboring terminals expressing VGLUT3 or mGLUR4. Scale bars: 300 μm in **(A)** (insets: 100 μm); 300 μm in **(I,K)**; 50 μm in **(J,L)**; 10 μm in K1-3 and L1–L2; 5 μm in L3. ^∗∗^*P* < 0.01, ^∗∗∗^*P* < 0.001.

As recently documented in the amygdala (Albayram et al., 2016), we found that VGLUT3-positive basket cells from the hippocampus neurons expressed CB1 endocannabinoid receptors (CB1R) (**Figure 5A**). Activation of CB1R strongly inhibits GABA release (Ferraguti et al., 2005). Hence, we investigated the status of CB1R in mice lacking VGLUT3 (**Figures 5A–D**). We found that neither CB1R transcripts (Mann–Whitney test, *P* = 0.4206, *U* = 8, **Figure 5B**), nor its binding sites (Mann–Whitney test, *P* = 0.3095, *U* = 7, **Figure 5C**) were modified in the CA1 pyramidal layer of VGLUT3^-/-^ mice. We next evaluated the effects of WIN55,212-2 (1 μM), a CB1R agonist, on GABA transmission. WIN55,212-2 inhibited GABA eIPSCs by 40 ± 8% in WT mice and by 45 ± 5% in VGLUT3^-/-^ mice, showing no significant alteration of CB1R signal transduction in the absence of VGLUT3 (Mann–Whitney test, *P* = 0.7209, *U* = 28, **Figure 5D**). These results demonstrate that VGLUT3-dependent inhibitory regulation of GABAergic transmission is independent of the CB1R signaling pathway.

Presynaptic group III mGLURs are found on GABAergic interneuron terminals. Their activation efficiently depresses GABA release onto CA1 pyramidal cells (Shigemoto et al., 1997; Fitzjohn et al., 1998; Somogyi et al., 2003). However, the source of endogenous glutamate responsible for this inhibition is unknown. We hypothesized that the increased GABA transmission observed in VGLUT3^-/-^ mice (see **Figures 3F,G, 4F,G**) was caused by the loss of an inhibitory control normally mediated by VGLUT3-dependent glutamate release and mGLUR activation. To test this hypothesis we investigated whether the broad-spectrum mGLUR antagonist LY341495 (75 μM) was able to stimulate GABAergic transmission (Shannon et al., 2005). In accordance with our hypothesis, the amplitude of eIPSCs was markedly increased by LY341495 in the WT mice (+61 ± 21%, *n* = 10, *P* < 0.01) but not in the VGLUT3^-/-^ mice (*n* = 11, Mann–Whitney test, *P* = 0.0002, *U* = 2, **Figure 5E**). Furthermore, LY341495 (75 μM) increased the frequency of spontaneous mIPSCs only in WT mice (+32 ± 8%, *n* = 11 compared with -3 ± 5%, *n* = 11 in VGLUT3^-/-^ mice, Mann–Whitney test, *P* = 0.0002, *U* = 19, **Figures 5F,G**) but had no effect on the amplitude of mIPSCs in both groups (0 ± 5%, *n* = 11 in WT group and 3 ± 7%, *n* = 11 in VGLUT3^-/-^ group, Mann–Whitney test, *P* = 0.7802, *U* = 41). Taken together these results show for the first time that VGLUT3-positive basket cells provide the glutamate necessary for pre-synaptic mGluR activation and the subsequent depression of GABAergic transmission onto CA1 pyramidal cells.

We then pharmacologically explored which subtype(s) of mGluR was (were) involved in this pathway (**Figure 5H**). Neither the group-I mGluR negative allosteric modulators LY456236 [500 nM (Gasparini et al., 1999)] and MPEP [10 μM (Kingston et al., 1998)] nor the group-II mGluR antagonist LY341495 [5 nM (Thomas et al., 1996)] had significant effect on eIPSCs amplitude in WT mice: 0 ± 7% (*n* = 6, Student *t*-test, paired, *P* = 0.7290, *t* = 0.37, df = 5) and +2 ± 11% (*n* = 9, Student *t*-test, paired, *P* = 0.5392, *t* = 0.64, df = 8), respectively. However, the group-III mGluR antagonist MSOP [300 μM (Kogo et al., 2004)] increased eIPSCs amplitude by 35 ± 10% (*n* = 8, Student *t*-test, paired, *P* = 0.0087, *t* = 3.60, df = 7). As expected, MSOP did not increase eIPSCs amplitude in VGLUT3^-/-^ mice (*n* = 7, Student *t*-test, paired, *P* = 0.8324, *t* = 0.22, df = 6). These results strongly suggest that VGLUT3-dependent glutamate inhibit GABAergic transmission by activating pre-synaptic group-III mGluRs.

**FIGURE 6:**
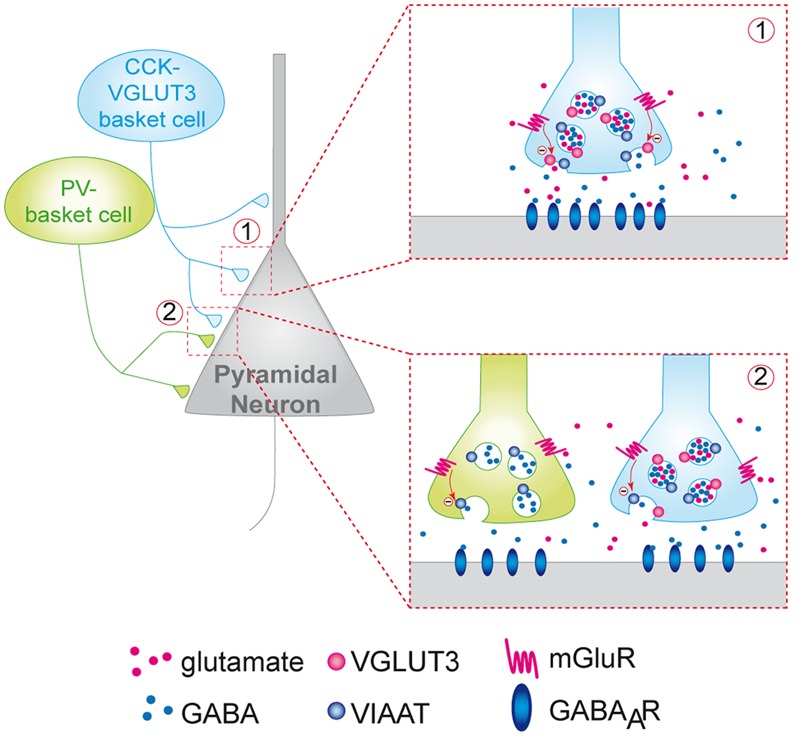
**Putative model of the inhibition of the release of GABA by VGLUT3-dependent glutamate and presynaptic mGLUR located on GABAergic terminals.** CCK/VGLUT3-positive basket cells (blue neuron) and PV basket cells (green neurons) establish a dense perisomatic network of terminals on pyramidal cells (gray neurons). Schematic representation of two models showing the inhibition of the release of GABA by VGLUT3-dependent glutamate: (1) Homosynaptic inhibition limited to CCK-VGLUT3 interneurons (blue, inset 1). Our results suggest that glutamate putatively released by CCK/VGLUT3-positive basket cells terminals could activate mGluRs (possibly group-III metabotropic glutamate receptors) located on the presynaptic side of the synaptic boutons. Their activation could inhibit the release from GABAergic synaptic vesicles release resulting in a decrease of the IPSC amplitude. (2) Heterosynaptic lateral inhibition by glutamate released from CCK/VGLUT3-positive basket cells (blue) of both CCK/VGLUT3- and PV-positive basket (green, left inset) according to the glutamate spillover model described previously (see Kullmann and Asztely, 1998).

To further confirm this hypothesis we examined whether mGLURs and VGluT3 were co-localized on perisomatic basket cell terminals in the CA1 pyramidal layer. Among the group-III mGluRs, mGluR4, mGluR7 and mGluR8 are known to be present on presynaptic elements of the hippocampus (Shigemoto et al., 1996; Fremeau et al., 2002; Somogyi et al., 2003). Using immunofluorescence, we found that mGluR4a (but not mGLUR7) was expressed in a subset of VGLUT3-immunopositive interneurons (**Figures 5I–L**). Indeed, some pyramidal cells were surrounded by VGLUT3- and mGluR4-positive boutons (**Figure 5L**). Observations along the *Z*-axis confirmed that VGLUT3 and mGluR4 were co-localized and not superimposed (**Figure 5L3**). Interestingly, most of the VGLUT3-positive boutons surrounding pyramidal cells co-expressed mGLUR4 (stars); however, in other cases, VGLUT3- and mGluR4-positive terminals were juxtaposed (arrow heads). Taken together, these results suggest that VGLUT3-containing terminals are physically co-localized or closely localized with mGluRs to modulate the release of GABA (proposed model **Figure 6**).

## Discussion

Using double FISH and immunofluorescent experiments we extend previous findings establishing that in the hippocampus, VGLUT3 is exclusively expressed in GABAergic interneurons (Gras et al., 2002; Herzog et al., 2004; Katona et al., 2006). In the hippocampus, a majority of CCK-positive basket cell terminals are CB1-positive (Hnasko et al., 2010; Lasztoczi et al., 2011; Albayram et al., 2016). In agreement with previous findings, we confirm that VGLUT3-positive interneurons are also CB1-positive (Hnasko et al., 2010; Albayram et al., 2016). Taken together our observations and previous publications suggest that VGLUT3 is present in CCK-positive baskets and more rarely in calbindin-positive interneurons in the hippocampus (Somogyi et al., 2004; Hnasko et al., 2010). Therefore, VGLUT3-CCK-positive basket cells (VCB) have the potential to release both glutamate and GABA, the main excitatory and inhibitory neurotransmitters in the brain, respectively.

Interestingly, only a minority of GABAergic interneurons and terminals expresses VGLUT3 (9–13%) in the CA1 region of the ventral hippocampus. Furthermore, VGLUT3 and VIAAT are present on the same synaptic vesicles, and VIAAT-immunopurified vesicles are able to accumulate [^3^H]glutamate. It has been shown that VGLUTs facilitate the storage of ACh, 5-HT and DA in vesicles, a phenomenon named vesicular synergy (Ferraguti and Shigemoto, 2006; Gras et al., 2008; Amilhon et al., 2010; Frahm et al., 2015). Data from the literature suggest that VGLUTs can also promote GABAergic transmission through vesicular synergy (Zander et al., 2010). Our finding that VGLUT3 ablation led to a 17% decrease in the amplitude of mIPSCs suggests that VGLUT3-dependent vesicular synergy could also operate in GABAergic basket cell terminals. However, increasing the load of GABA into synaptic vesicles is not the prevailing effect of VGLUT3 on GABAergic synaptic transmission.

In this study we revealed a new VGLUT3-dependent glutamatergic modulation of GABAergic transmission through an autoreceptor-mediated presynaptic mechanism (**Figure 6**). Indeed, one of the most striking observation was the marked increase in GABAergic transmission onto CA1 pyramidal cells in the VGLUT3^-/-^ mice strain. Using a VGLUT3^V IAAT-Cre-flox/flox^ mouse line with a specific ablation of VGLUT3 in GABA neurons, we further confirmed that this effect is indeed related to the expression of VGLUT3 in GABAergic neurons.

Moreover, we also found a significant increase in the frequency of unitary IPSCs (+76%) in the VGLUT3^-/-^ mice and an increase in monosynaptic-evoked IPSCs amplitude in both VGLUT3^-/-^ (+68%) and VGLUT3^V IAAT-Cre-flox/flox^ (+55%) mice. Thus, despite their low abundance, VGLUT3-positive terminals exert a robust inhibitory control on the local release of GABA. Previous anatomical and pharmacological data have shown that group III mGLUR4, mGLUR7 and mGLUR8 can be found on GABAergic terminals in the hippocampus (for review, see Semyanov and Kullmann, 2000). The leading theory explaining the role of mGluR activation was that principal neurons or astrocytes might be the source of glutamate that binds to mGluRs and regulates GABA release (Kullmann and Asztely, 1998). Here, we present evidence that mGluR4 are present on VGLUT3-positive terminals and on the perisomatic terminals of other neighboring basket cells. From a mechanistic point of view, our results suggest that VCB cells are a likely important source of the glutamate that activates these mGluRs (**Figure 6**). Furthermore, it is noticeable that 10% of terminals can inhibit 70% of the local GABAergic transmission. One speculative explanation could be that VCB synapses may exert inhibitory control over neighboring GABAergic terminals in a spillover phenomenon (see Ren et al., 2011 and **Figure 6** inset 2).

As for the VGLUT3-dependent postsynaptic actions, data from the literature have shown that the expression of a VGLUT also provides modulatory neurons with the ability to mediate fast, glutamatergic, AMPA receptor-mediated postsynaptic depolarizations (Ferraguti and Shigemoto, 2006; Karson et al., 2009; Varga et al., 2009; Higley et al., 2011). Previous studies using paired recording experiments have not described the occurrence of glutamatergic-dependent postsynaptic responses in pyramidal cells or in PV interneurons in response to CCK interneurons stimulation (Losonczy et al., 2004; Colgin, 2013); however, it was unclear whether the CCK interneurons evaluated in these studies expressed VGLUT3. Therefore, it remains unclear whether VCB cells can directly depolarize postsynaptic neurons following the release of glutamate.

With respect to synaptic plasticity and network activity, the disinhibition of hippocampal GABAergic transmission associated with the absence of VGLUT3 altered the oscillatory activity of synchronized networks and induced a metaplastic shift of synaptic plasticity in the ventral hippocampus. The frequency of theta oscillations was significantly reduced in the VGLUT3^-/-^ mice. Hippocampal theta oscillations are thought to contribute to learning and memory and the expression of anxiety in rodents (Hasselmo et al., 2002; Gordon et al., 2005; Jutras et al., 2013). Many theories suggests that coordinated activity of hippocampal neurons in the frequency range of 4–8 Hz is implicated in the separation between encoding and retrieval of memory traces trough the oscillation phase-locking with either entorhinal inputs or CA3 inputs at CA1 pyramidal neurons (Huerta and Lisman, 1995). Consequently, phasic changes in synaptic transmission induced by theta oscillations have been proposed to regulate the gating of bidirectional synaptic plasticity at CA1 pyramidal neurons (Huerta and Lisman, 1995). The relationship between local peaks and sinks of theta waves and LTP and LTD could particularly be observed on slice preparation when theta rhythm was induced by cholinergic agonists (Seal et al., 2008). Consequently, any shift or alteration in the oscillatory properties of pyramidal neurons could induce perturbations of synaptic plasticity mechanisms. These changes could also lead to an alteration of learning and memory processes accompanied or not with an impaired expression of anxiety. In line with these observations an increased anxiety was previously reported in VGLUT3 null mice (Amilhon et al., 2010).

It has been reported that constitutive deletion of VGLUT3 results in generalized epilepsy with very little change of motor behavior (Boyce et al., 2016). It can therefore not be ruled out that some of the hippocampal phenotypes herein reported (such as theta oscillation modifications) could result from these non-convulsive seizures in VGLUT3^-/-^ mice. However, the ≈50% increased GABA tone should on the contrary tone down epileptic activity. These conflictual observations will deserve future investigation.

We observed that the disinhibition of hippocampal GABAergic transmission associated with VGLUT3 loss altered the metaplastic properties of the hippocampal network. In VGLUT3^-/-^ mice, spontaneous theta oscillations of ventral CA1 significantly shifted to lower frequencies. They were accompanied by a different profile of synaptic plasticity in ventral slice preparation. Under classical conditions, low frequency stimulations (1 to 10 Hz) generally induce LTD of fEPSPs at Schaffer-CA1 synapses. As expected, both stimulations at 1 and 3 Hz induced significant LTD in WT mice. However, in VGLUT3^-/-^ mice low frequency stimulation at 1 Hz induced LTD but not at 3 Hz (the frequency of spontaneous theta oscillations we observed *in vitro*). This is in accordance with the previously mentioned theory that stimulation of Schaffer’s collaterals in a frequency range close to the theta synchronicity of the network would rather be in favor of potentiation. In hippocampus slice preparations at Schaffer-CA1 synapses, LTP is induced by protocols using a high-frequency stimulation (50–100 Hz). In the absence of VGLUT3, tetanic HFS-induced LTP was markedly absent. However, when GABA_A_ blockers were used to inhibit fast GABAergic transmission, LTP was restored to control level. Interestingly, similar expression of TBS-induced LTP was observed in WT and VGLUT3^-/-^ mice slices. TBS is composed of both theta and tetanic high frequency components. We hypothesize that while at high frequencies of stimulation, the strong disinhibition of GABAergic transmission in VGLUT3^-/-^ mice prevails on postsynaptic excitability – an effect that is abolished with the GABA-A antagonist bicuculline – the 5 Hz component of TBS still enables VGLUT3^-/-^ mice synapses to undergo sufficient potentiation to express significant LTP. Taken together, the field recording results show that VGLUT3^-/-^ mice still express a significant amount of bidirectional synaptic plasticity but the frequency-plasticity relationship in VGLUT3^-/-^ mice appears shifted toward the lower frequencies of stimulation. The implication of VGLUT3 in such metaplastic shifting could have complex but significant effects on hippocampal related processes such as learning and memory.

Our data suggest that the VGLUT3-dependent release of glutamate by hippocampal interneurons could finely tune GABAergic transmission onto principal cells and modulate metaplasticity at glutamatergic synapses. If our working hypothesis depicted in **Figure 6** is correct, this tuning could be mediated by a presynaptic mechanism involving the activation of type III mGluR autoreceptors. The long-term plasticity of hippocampal synapses is currently thought to underlie the cellular basis of learning and memory (Cooke and Bliss, 2006; Omiya et al., 2015). Among their many other functions, theta oscillations are thought to contribute to learning and development of anxiety in rodents (Hasselmo et al., 2002; Gordon et al., 2005; Jutras et al., 2013). Therefore, it can be speculated that VGLUT3 could play a key role in emotion-associated memories in the ventral hippocampus.

## Author Contributions

CF, SW, and SEM designed the study and wrote the manuscript with inputs from BG, GA-H, SD, JR, and KP. CF, JR, KP, MM-S, RG, and FM performed electrophysiology experiments and analyzed the data. CF, DS, MM-S, DC, LR, and SB performed immunohistochemistry experiments and analyzed the data. VB performed electron microscopy experiments. SD designated oligonucleotides and designed *in situ* hybridization protocols. EV performed *in situ* hybridization, double FISH and analyzed the data. J-FZ performed Immuno-isolation of synaptic vesicles, western blot and [^3^H]glutamate uptake assay and analyzed the data. LM performed autoradiography experiments.

## Conflict of Interest Statement

The authors declare that the research was conducted in the absence of any commercial or financial relationships that could be construed as a potential conflict of interest.

## Footnotes

1 http://ccac.ca/en_/standards/guidelines

2 http://www.phenomin.fr/
