# Supplementary material for: Regulation of the Hippocampal Network by VGLUT3-Positive CCK- GABAergic Basket Cells
Source: Front Cell Neurosci. 2017 May 16;11:140. doi: 10.3389/fncel.2017.00140 (PMC5432579; doi:10.3389/fncel.2017.00140)
Supplement: Supplementary file 1 [file Presentation_1.PDF]

SUPPLEMENTAL INFORMATION

Regulation of the hippocampal network by VGLUT3 and glutamate released from GABAergic basket cells.

Caroline Fasano, Jill Rocchetti, Katarzyna Pietrajtis, Johannes-Friedrich Zander, Frédéric Manseau, Diana Yae Sakae, Maya Marcus-Sells, Lauriane Ramet, Lydie Jacqueline Morel, Damien Carrel, Susanne Bolte, Véronique Bernard, Erika Vigneault, Romain Goutagny, Gudrun Ahnert-Hilger, Bruno Giros, Stéphanie Daumas, Sylvain Williams and Salah El Mestikawy

Recorded GABAergic IPSCs are characterized by an inward current at resting potential caused by a high chloride concentration in the intracellular solution.

The Cl^- reversal potential was set at -16 mV to detect GABAergic IPSCs at -70 mV. Under these conditions, monosynaptically elicited eIPSCs in a pyramidal neuron (**Fig. S1A**) held at -70 mV were represented by an inward current (**Fig. S1B**). Fast glutamatergic transmissions were blocked with DNQX and AP5 for all patch-clamp recordings. The perfusion of bicuculline (5 µM) completely abolished the recorded inward current, which confirmed the GABAergic nature of the postsynaptic events. An inhibitory square pulse was applied before each stimulation to measure the input resistance and assess the quality of the patch. Recordings were excluded if the input resistance varied more than 20 %. Although the theoretical ECl^- was estimated to be approximately -16 mV by the Nernst equation, the measured ECl^- was approximately -8 mV on the IV curve of GABAergic currents (n = 5, **Fig. S1C**).

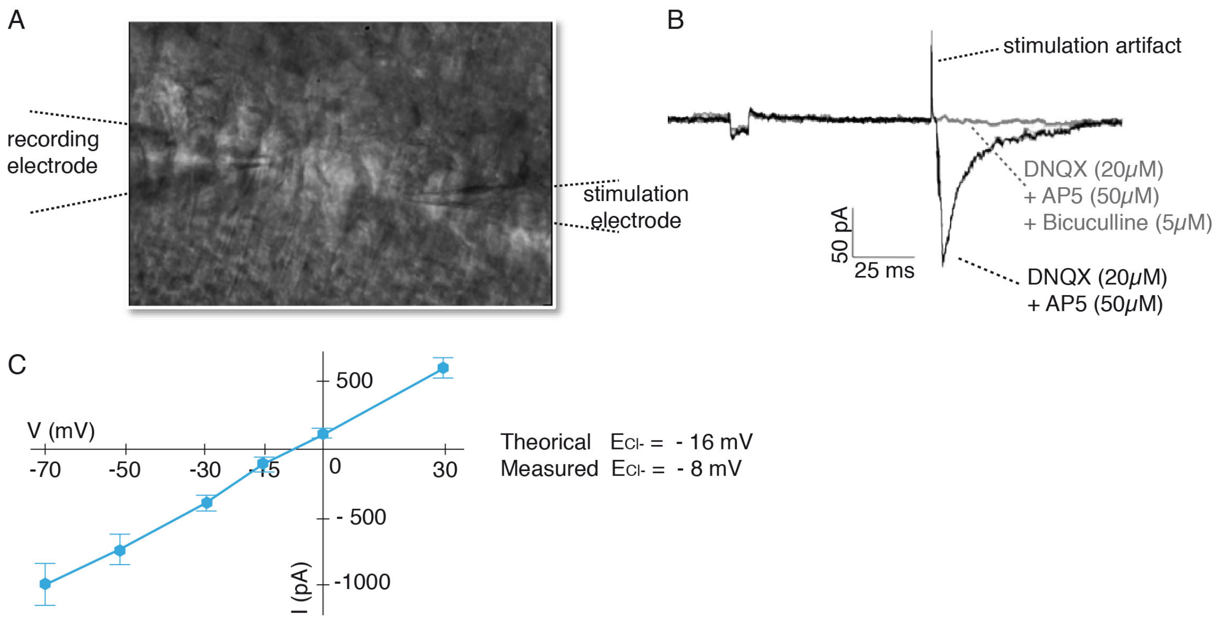

Supplemental Figure S1**: Patch-clamp recording of GABAergic IPSCs with a high chloride-containing intrapipette solution.** (**A**) Photomicrograph of a hippocampal section during a recording taken with a 40x water-immersion objective (Olympus, Canada) showing the location of the recording electrode and the stimulation electrode inducing monosynaptically evoked IPSCs in the CA1 stratum pyramidale. (**B**) Example of an eIPSC recording at -70 mV during the blockade of fast glutamatergic transmission with DNQX and AP5. The stimulation induced an inward current (black trace) that was abolished in the presence of bicuculline (5 µM $GABA_A$ receptor antagonist, gray trace). (**C**) IV curve of GABAergic eIPSCs.

Supplemental Table S1: **Glutamate uptake measured on immunoisolated glutamatergic (VGLUT1-positive) or GABAergic (VIAAT-positive) vesicles.**

**test**	**n**	**Student t-test, unpaired, 2-tailed**	
**(Fig. 1I)** Vesicle Immunoisolation	VIAAT = 4<br>IgG = 4	*VIAAT vs IgG*	
		***P*-value**	**t-value**
		P = 0.0028 **	*t=2.65*

Principle was proven in Zander et al. 2010

**: $P < 0.01$

Supplemental Table S2: **VGLUT3 regulates network activity and metaplasticity of the hippocampus.**

**test**	**n**	**time**	**Mann Whitney test**
**(Fig. 2B)** theta oscillations	WT = 12<br>VGLUT3$^{-/-}$ = 14	5-10min	*U = 9.5, P* = 0.0002 ***
		95-100min	*U = 29.5, P =0.0054* **

**test**	**n**	**Wilcoxon test (baseline vs. response)**			**Mann Whitney test (WT vs VGLUT3$^{-/-}$)**
		***P* value**	**W value**	**n of pairs**	
**(Fig. 2C)** HSF	WT = 9	*P* = 0.0039**	W = -45	9	*U = 19 P* = 0.0350*
	VGLUT3$^{-/-}$ = 10	*P* = 0.4922	W = -15	10	
**(Fig. 2D)** HFS + Bicuculline	WT = 6	*P* = 0.0313*	W = -21	6	*U = 11, P* = 0.3095
	VGLUT3$^{-/-}$ = 6	*P* = 0.0313*	W = -21	6	
**(Fig. 2E)** TBS	WT = 9	*P* = 0.0039**	W = -45	9	*U = 36 P* > 0.9999
	VGLUT3$^{-/-}$ = 8	*P* = 0.0078**	W = -36	8	
**(Fig. 2F)** LFS-1Hz	WT = 6	*P* = 0.0313*	W = 21	6	*U = 18 P* = 0.3153
	VGLUT3$^{-/-}$ = 9	*P* = 0.0195*	W = 39	9	
**(Fig. 2G)** LFS-3Hz	WT = 7	*P* = 0.0313*	W = 26	7	*U = 8 P* = 0.0379*
	VGLUT3$^{-/-}$ = 7	*P* = 0.2969	W = 14	7	

*: $P < 0.05$, **: $P < 0.01$, ***: $P < 0.001$.

Supplemental Table S3: **VGLUT3 regulates GABAergic but not glutamatergic synaptic transmission.**

Test	n	RM-ANOVA
(**Fig. 3A**) Input/Output	WT = 10<br>VGLUT3$^{-/-}$ = 10	main effect of genotype F(1,18) = 0,1799 P = 0.6765

Test	n	Fig.	Mann Whitney test
(**Fig. 3 C and E**) mIPSCs	WT = 27 VGLUT3$^{-/-}$ = 25	Fig 3C, peak amplitude	*U = 210, P* = 0.0200 *
		Fig. 3E, frequency	*U = 217.5, P* = 0.0438 *
(**Fig. 3F**) eIPSC	WT = 16 VGLUT3$^{-/-}$ = 14	Fig. 3F, sigmoid plateau	*U = 63, P* = 0.0151*

*: $P < 0.05$.

Supplementary Table S4**: Quantification of VGLUT3 expression in the hippocampus of VGLUT3$^{\text{VIAAT-Cre-flox/flox}}$ mice.**

**test**	**Two-way ANOVA (genotype)**		**Bonferroni post-tests**		
	***P* value**	**F value**	**groups**	**t value**	***P value***
(**Fig. 4D**) IAR VGLUT3 WT, n = 5 cKO, n = 5	*P* < 0.0001***	F (5,48) = 68.15	total hippocampus	2.792	P < 0.05 *
			total CA1	3.363	P < 0.01 **
			CA1 - Strata Oriens	2.914	P < 0.05 *
			CA1 - Strata Pyramidale	9.197	P < 0.001 ***
			CA1 - Strata Radiata	1.456	P > 0.05
			CA1 - hippocampus fissure	0.499	P > 0.05

**test**	**n**	**Fig.**	**Mann Whitney test**
(**Fig. 4F and G**) eIPSC	WT = 10 cVGLUT3$^{-/-}$ = 10	Fig. 4F and E, sigmoid plateau	Amplitude : *U = 21, P =0.0028**

*: *P* < 0.05, **: *P* < 0.01, ***: *P* < 0.001.
cVGLUT3$^{-/-}$ = VGLUT3$^{\text{VIAAT-Cre-flox/flox}}$ mice

Supplementary Table S5: **VGLUT3-dependent regulation of GABAergic synaptic transmission is not operated by CB1-R but by type-III mGLURs.**

test	n	Fig.	Mann Whitney test
(**Fig. 5B-D**) CB1	WT = 5 cVGLUT3$^{-/-}$ = 5	Fig. 5B, ISH CB1	number of nuclei: *U = 8, P =0.4206*
	WT = 5 cVGLUT3$^{-/-}$ = 5	Fig. 5C, Binding CB1	intensity: U= 7, *P* = 0.3095
	WT = 8 cVGLUT3$^{-/-}$ = 8	Fig. 5D, eIPSC	Amplitude: U = 28, *P = 0.7209*
(**Fig. 5E, F**) IPSC and mGluRs	WT = 10 VGLUT3$^{-/-}$ = 11	Fig. 5E, eIPSC	Amplitude: *U = 2, =0.0002 ****
	WT = 11 VGLUT3$^{-/-}$ = 11	Fig. 5F, mIPSC	Frequency: U= 19, *P* = 0.0071 **
			Amplitude: U = 41, *P = 0.7802*

test	Type-specific blocker	n	Paired t-test (baseline vs drug effect)		
			***P* value**	**t value**	**df**
(**Fig. 5H**) IPSC and mGluRs	Type 1, 2, 3	WT = 10 VGLUT3$^{-/-}$ = 10	*P* = 0.0005***	t = 5.26	df = 9
			P = 0.3155	t = 1.06	df = 9
	Type 1	WT = 6	*P = 0.7290*	t = 0.37	df = 5
	Type 2	WT = 9	*P = 0.5392*	t = 0.64	df = 8
	Type 3	WT = 8	*P* = 0.0087*	t = 3.60	df = 7
		VGLUT3$^{-/-}$ = 7	*P = 0.8324*	t = 0.22	df = 6
	**One-way ANOVA (all groups)**		**Tukey's Multiple Comparison test**		
	***P* value**	**F value**	**groups**	***P* < 0.05 ?**	
	P < 0.0001	F = 7.103	Type 1,2,3 WT vs Type 1,2,3 VGLUT3$^{-/-}$	yes	
			Type 1,2,3 WT vs Type 3 WT	no	

*: *P* < 0.05, **: *P* < 0.01, ***: *P* < 0.001.
cVGLUT3$^{-/-}$ = VGLUT3$^{VIAAT\text{-}Cre\text{-}flox/flox}$ mice
